# Lessons learned from setting up the Nahuche Health and Demographic Surveillance System in the resource-constrained context of northern Nigeria

**DOI:** 10.3402/gha.v7.23368

**Published:** 2014-05-07

**Authors:** Olatunji Alabi, Henry V. Doctor, Godwin Y. Afenyadu, Sally E. Findley

**Affiliations:** 1 Nahuche Health and Demographic Surveillance System and the PRRINN-MNCH Programme, Kano, Nigeria; 2 United Nations Office on Drugs and Crime, Country Office for Nigeria, Abuja, Nigeria; 3 Department of Population and Family Health, Mailman School of Public Health, Columbia University, New York, USA

**Keywords:** maternal and child health, demographic surveillance, health systems, INDEPTH Network, Nigeria

## Abstract

**Background:**

The present time reflects a period of intense effort to get the most out of public health interventions, with an emphasis on health systems reform and implementation research. Population health approaches to determine which combinations are better at achieving the goals of improved health and well-being are needed to provide a ready response to the need for timely and real-world piloting of promising interventions.

**Objective:**

This paper describes the steps needed to establish a population health surveillance site in order to share the lessons learned from our experience launching the Nahuche Health and Demographic Surveillance System (HDSS) in a relatively isolated, rural district in Zamfara, northern Nigeria, where strict Muslim observance of gender separation and seclusion of women must be respected by any survey operation.

**Discussion:**

Key to the successful launch of the Nahuche HDSS was the leadership's determination, stakeholder participation, support from state and local government areas authorities, technical support from the INDEPTH Network, and international academic partners. Solid funding from our partner health systems development programme during the launch period was also essential, and provided a base from which to secure long-term sustainable funding. Perhaps the most difficult challenges were the adaptations needed in order to conduct the requisite routine population surveillance in the communities, where strict Muslim observance of gender separation and seclusion of women, especially young women, required recruitment of female interviewers, which was in turn difficult due to low female literacy levels. Local community leaders were key in overcoming the population's apprehension of the fieldwork and modern medicine, in general. Continuous engagement and sensitisation of all stakeholders was a critical step in ensuring sustainability. While the experiences of setting up a new HDSS site may vary globally, the experiences in northern Nigeria offer some strategies that may be replicated in other settings with similar challenges.

This is a period of intense effort to get the most out of public health interventions, with an emphasis on health systems reform and implementation research. In order to determine which health systems reforms and programmatic combinations are better at achieving the goals of improved health and well-being, we need the capacity to implement pilots of promising interventions in the ‘real world’ (1–4). Further, this research needs to be time sensitive, with findings returned to decision makers in a relatively short time period, that is months, not years. Population surveillance sites offer a solution to this problem, but operating these sites entails a strong and permanent research capacity. Ironically, precisely where the studies are needed the most are the locations where it is most difficult to assemble and support such a team. The challenge then is how to operationalise a population surveillance site which can handle complex design issues in the face of limited research and management infrastructure.

Nigeria is one of the countries facing this dilemma. Northern Nigeria has some of the poorest maternal and child health outcomes in the world, with only 21% of pregnant women receiving antenatal care in their last pregnancy and a child mortality rate of 246 per 1,000 in 2008. The primary health care (PHC) system was desperately in need of refurbishment and reform after years of neglect. With funding from UK Aid (Department for International Development) and the Norwegian government, the Partnership for Reviving Routine Immunisation, Maternal, Newborn, and Child Health (PRRINN-MNCH) was established in 2008 to implement comprehensive health system improvements (5). State governments were eager to implement a host of improvements throughout the primary care system, but decision makers were hampered by a paucity of timely information about how they impact health behaviours and outcomes in their particular settings. When these data are available, such as the Nigerian Demographic Health Surveys of 2008 and 2013, they do not provide the level of detail needed to assess the different impacts of alternative programme interventions. A completely different design was needed to support decisions about competing programme options.

Population health approaches provide a ready response to this problem. A stream of publications from population surveillance sites reflects the match between population health approaches and the health systems research approach (6–8). Given the need to compare alternative programme intervention models using a population health perspective, PRRINN-MNCH undertook to launch a Health and Demographic Surveillance System (HDSS) site.

The INDEPTH Network is known for its pioneering leadership in health and population research through its global network of HDSS field sites in Africa, Asia, and Oceania. The INDEPTH Network is capable of producing reliable longitudinal data about the lives of people across the three continents and has assisted many countries in setting up HDSS sites. INDEPTH is a rural model, with very few urban sites. This paper describes and shares our experience in successfully setting up an HDSS which successfully met these criteria and became a member of the INDEPTH Network.

## Methods

### Why Nahuche and the vision for Nahuche HDSS: PRRINN-MNCH Programme

The first step in establishing an HDSS was to select the site. Two state governments were interested in housing the site: Jigawa and Zamfara. To determine which state (if any) and which site within the state met the criteria for an HDSS, PRRINN-MNCH hosted a scoping visit by a team of international researchers with experience in developing and collaborating with INDEPTH sites. The purpose of the visit was to assess their understanding and commitment of each state's stakeholders to the establishment of an HDSS, and then through discussions and site visits assessing the extent to which the potential sites met the requisite criteria for an HDSS site. The criteria for an HDSS site include: minimum of 40,000 population size; uninterrupted internet service; stable electricity supply (which may be achieved through alternative backups like standby generators and inverters); access to skilled labour force, e.g. computer specialists, programmers, interviewers; access to a diverse population who can provide different responses and a population that wants answers and is willing to answer questions to help researchers find solutions to problems; ability to interface with state and federal planners to identify health system research combinations to study and ability to mobilise resources to sustain the operation of the site among others.

In each state, the international team first met with several stakeholders, including PRRINN-MNCH Programme officials, members of the State Health and Planning Ministries, local government and community leaders in potential sites. They conducted group discussions with community, local government, and state officials and assessed the acceptability of the initiative, ongoing efforts in population registration and commitment to population-based research on health outcomes, among others. The availability of supportive infrastructure such as suitable housing and offices was also discussed with the state government officials. The scoping mission next surveyed the physical and social characteristics of the proposed HDSS community sites to ensure that they had sufficient population size and accessibility to interviewers. They also identified existing health systems structures and initiatives in the states, and in the local governments proposed for the HDSS sites in each state.

While sites in both Zamfara and Jigawa met the criteria in terms of population size and supportive infrastructure, the state and local government support among stakeholders was found to be much stronger in Zamfara. Zamfara, particularly the Bungudu Local Government Area and the district of Nahuche, offered housing and infrastructure support. Further, the Nahuche site included a functional PHC where various PRRINN-MNCH Programme interventions would be based. Therefore, the team recommended that Nahuche District in Zamfara be selected for the development of the Nahuche site.

To ensure their understanding and commitment, key PRRINN-MNCH implementation research leaders and Nahuche stakeholders were invited to visit the HDSS site of Navrongo Health Research Centre in northern Ghana, a fairly comparable setting. The tour included field visits and presentations on the role of research in health systems strengthening and service delivery and translation of research findings into action. Throughout this visit, the PRRINN-MNCH team learned first-hand from the Navrongo experience and was able to solicit technical support and collaboration on setting up the Nahuche HDSS.

### The study area

**Fig. 1:**
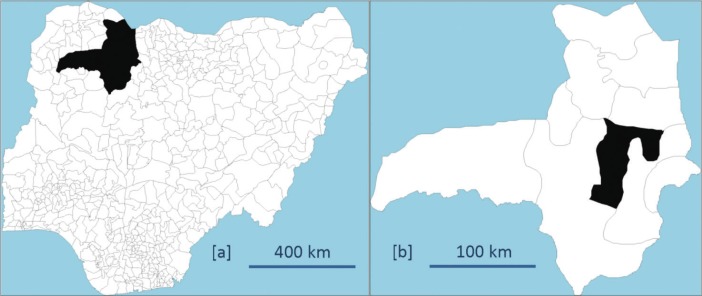
(a) Map of Nigeria showing Zamfara State and (b) Zamfara State showing Bungudu Local Government Area.

The Nahuche HDSS study site with coordinates 12°36′ N, 6°54′ E, and 435 m above sea level is located in Zamfara State of north-western Nigeria (Fig. 1). Nahuche has a typical Sahelian climate with temperatures reaching a high of 38°C from March to May and a rainy season from May to late September, while the cold season, the Harmattan, starts from December until February. Situated in Bungudu LGA, the site is 32 km from the state capital, Gusau, and constitutes six districts of Bella, Gada, Karakai, Nahuche Keku, Nahuche Ubandawaki, and Rawayya. Gusau is a key commercial centre with a heterogeneous population from all over Nigeria.

The Nahuche HDSS study site is made up of 120 villages under the leadership of six district heads. Almost all residents in the study area are Hausa. Farming is the most common economic and subsistence activity of the people and is consistent with the slogan of the state ‘farming is our pride’ (9). High unemployment is a catalyst for temporary labour migration among men. The standard of living is quite low, with hardly any families owning cars or televisions, standard signs of improved living standards. Infrastructure remains deficient with no access to an electricity grid and dependence on local generators. There is no community sanitation system, and most families are depending on latrines and wells or boreholes for their water. All villages have at least a primary school, and some have a junior secondary school. One primary health centre is located adjacent to the HDSS site offices while the general hospital is located on the way to Gusau. Most people access healthcare from both Traditional healers and faith based healers apart from public health facilities.

### Stakeholder participation: the implementation of steering committee

On 12 October 2009, a steering committee was set up to fast track the activities that would ultimately lead to a successful establishment of the Nahuche HDSS. Membership of the steering committee was drawn from relevant government agencies including the Ministries of Health and Planning and Budget, Primary Health Care Development Agency, the Primary Health Care (PHC) directorate of the Bungudu LGA, State Ministry of Local Government (MOLG), and the Zamfara State Team Leader for PRRINN-MNCH. The committee's mandate was to mobilise resources for the HDSS set-up and to facilitate collaboration with communities in the catchment area and with the Bungudu LGA. The state and LGA were asked to recruit junior cadre staff to facilitate the launch of the site, nominate members to the operations research advocacy committee (ORAC), which would serve as the ethics review board for the HDSS. The ethics sub-committee of the ORAC works as an Internal Review Board, and would review all research proposals implemented on the HDSS platform, as well as any in the state. All work of the committee was voluntary.

Community mobilisation and collaboration is key in longitudinal surveillance of a population. The Nahuche HDSS Steering Committee began the community mobilisation by ensuring high-level commitment by officials from the State Ministry of Health and other state government departments. The committee then led the community mobilisation through repeated visits and briefings with the district heads and other community leaders. Continuing as a part of the ongoing activities of the HDSS, the community mobilisation meetings are organised in each district by two *Imams* from the two Islamic groups and two other community members appointed by the chief and the officer-in-charge of the PHC from each district. During the community meetings, issues discussed included: the purpose of the HDSS establishment, and support and commitment of the people and the traditional leaders were solicited.

### Resource mobilisation

A key part of establishing the HDSS was the memorandum of understanding (MoU) signed between PRRINN-MNCH and the Zamfara State government. The MoU clearly spelt out the resource mobilisation guidelines for the HDSS centre. Three major partners were identified and each with its financial obligations: 1) Bungudu LGA provided three blocks of flats for use as office accommodation for the centre and was responsible for the perimeter fencing of the centre. 2) PRRINN-MNCH Programme provided technical and financial assistance for the successful implementation of the project. In addition, PRRINN-MNCH provided a four-wheel drive vehicle, office furniture, electric generator, motorcycles, computers with battery backups, internet service, and payment of salaries. 3) The Zamfara State government was asked to pass a law designating the HDSS as an agency of state with a budgetary allocation to ensure secured sustainable funding of the centre. The State Ministry of Health also was expected to provide senior staff (a demographer, a statistician, a public health expert, and an administrator) to work alongside the three long-term consultants employed by PRRINN-MNCH Programme for the HDSS; however, they were unable to provide this senior technical staff.

As a result, the HDSS steering committee forged a strategic alliance between Zamfara State government and Usman Danfodiyo University, Sokoto (UDUS), which had several faculties able and willing to provide the necessary technical expertise. Therefore, an MoU was subsequently signed between the state government and UDUS in order to harness the technical resources available in the university and to ensure sustainability of the centre beyond the programme.

### Recruitment and training of technical and field staff

With guidance from the INDEPTH Network and PRRINN-MNCH senior technical advisors on job specification and qualifications, advertisements were placed in the public media and persons were recruited for the positions of the HDSS manager, computer scientist, and field data managers. The INDEPTH Network helped train the PRRINN-MNCH technical advisor and the successful candidates at the Navrongo HDSS site in November 2009 to prepare them for set-up and the pilot census. Through contractual arrangements that mapped specific areas of need for technical assistance, INDEPTH provided a database management expert and an HDSS field management expert to give intermittent follow-on guidance and training to the data manager, HDSS manager, and the enumerators for the pilot census, baseline census and subsequent four rounds of data collection.

### The field staff

The quality of HDSS data depends to a large extent on the quality and commitment of the field staff. Getting adequately qualified persons in the surrounding communities was an uphill task. Literacy levels were generally low in these deprived rural communities but even lower among women. Being predominantly Muslim communities, men are not allowed to interview females who are socio-culturally subjugated, so it was imperative that female interviewers were recruited. This meant that the recruitment of field staff had to do extraordinary outreach to find and recruit suitable female interviewers. Even though the screening and recruitment of fieldworkers and data entry clerks was an open selection process through advertisement of vacant positions for qualified candidates to apply, particular attention was given to any women who applied. In fact, few women from the LGA and local communities applied for the job. Further, most of them failed the screening test due to their lower literacy level. Therefore, in order to recruit females, females who demonstrated potential to succeed even if their scores were lower than those of men were selected.

Fieldwork and data entry training manuals were developed using samples from other HDSS sites. All those who passed the initial screening for literacy, residency in the surveillance zone, aptitude for interviewing (or data entry) were trained. The fieldworkers and data entry clerks were trained together to ensure that the latter understood the nature of the data to be collected. In this way, fieldworkers would understand the structure of the data entry processing software and appreciate the built-in functionality of the interview to provide for consistency checks. The interviewers could see how any mistakes or inconsistencies contained in the completed questionnaire could be detected.

Those with the highest scores in the post-test training were hired as field workers and data entry clerks, with the exceptions noted above to give priority to promising female interviewers who did not do as well on the written test. While 250 candidates came for the recruitment screening interviews, 50 applicants barely met the minimum criteria for selection. Out of the 50 applicants who were trained, only 25 were finally selected (20 fieldworkers and five data entry clerks). Out of the 20 fieldworkers, only four were females whereas out of the five data entry clerks, only one was female.

### Launching the HDSS: baseline census

The first step in establishing the database that is to become the surveillance system is to conduct a baseline census, in which the surveillance area is mapped, all communities and households or compounds are mapped, the area is subdivided or chunked into manageable surveillance units (to include about 500 persons each), and then the team conducts a census to establish the number and characteristics of the surveillance population at the initial baseline. Our first step in this process was to send a team of HDSS staff from Nahuche to the Navrongo HDSS site from 22 November to 6 December 2009 to learn all of the critical processes for rolling out an HDSS.

### Pilot mapping, compound numbering, and coordinate taking

Researchers, policymakers, and programme managers have long recognised geographic location as an important factor in population and health outcomes. Knowing how the health of individuals differs by place of residence can lead to a better understanding of where and why events occur and how interventions can be implemented effectively. The Nahuche HDSS therefore included geographic positional coordinates in its mapping of communities and households within the Nahuche surveillance site.

Mapping, compound numbering, and recording of global positioning system (GPS) coordinates were done in collaboration with the Nigeria's National Population Commission (NPopC), which has the mandate and resources to lead demarcation and other activities for census taking in Nigeria. The regional NPopC staff directed the mapping of the surveillance area and its demarcation into smaller units (clusters) for easy enumeration, by re-demarcating the current Enumeration Area (EA) census maps from the NPopC into smaller units referred to as clusters. Each cluster was demarcated to include about 500 people in approximately 72 compounds. A compound was defined as a dwelling unit or structures comprising of one or more households. The staff were grouped into four teams with NPopC staff and some community leaders to identify the boundaries of the clusters and note key features on the cluster maps.

As is common throughout INDEPTH sites, it is important that the interviewers know exactly which household they are visiting, so that the interview results can be entered for the correct household, round after round. Therefore, compounds have a publicly visible number painted on their exterior compound wall. These numbers were designed to match up with the identification code of the household in the database, making it easier for the interviewer to ensure that they are speaking with the correct household. The compound codes identify the Nahuche EA or district, cluster, and compound. On the interviewer's census form, this code would identify the compound, and the interviewer would add codes for household and member within the household. For example, NKA001001 denotes the first household in the first compound of the A cluster of the Nahuche Keku District.

When the first round of GPS coordinates were mapped, the NPopC staff went with the field team to re-take the coordinates of a sample of the compounds in each cluster as a quality assurance measure. Re-taking coordinates not only assured the quality of the data but also provided an opportunity to retrain some staff who were still struggling with the appropriate use of the GPS equipment.

### Implementing the pilot census

With approximately 120,000 residents, the baseline census would be an enormous undertaking. Therefore, to ensure that the team was adequately prepared and all details managed, we conducted a pilot census in the 39 villages of Nahuche Keku District (EA), May–June 2010. The HDSS field management team employed a team approach to fieldwork, which generally consisted of one team leader with five or six field staff. For convenience and based on the available team leaders, a total of 25 field staff were divided into four groups, one per cluster.

At the pre-census refresher training, fieldworkers were trained in completing the questionnaires and in particular how to ask the questions so that they obtained accurate and complete answers. If an interview was not completed on the first visit, further attempts were made with the household or respondent, up to three times and over three different days, before classifying the case as non-contact. The subsequent contacts were scheduled at times when the respondent was more likely to be at home. The team leader or supervisor reviewed all completed interviews daily, checking thoroughly for empty response fields, illegible responses, and inconsistent responses, using the internal consistency checks built into the questionnaire. Fieldworkers were provided with feedback before proceeding to collect data on the following day.

Between May 25 to June 2, 2010, 1,456 individuals were enumerated from 197 households in the four clusters. The results and findings from the pilot study have been documented elsewhere (9) and informed the roll-out of the baseline census. The HDSS programme manager provided additional refresher training on the completion of the items, and in particular on the complete listing of all members of the compound, even those absent on the day of the interview. The number of households per interviewer per day was reduced from five to four. In addition, the interviewers were given better guidelines for responding to community requests for medical help or other benefits in appreciation for participating in the census. Finally, the supervisory process was better organised, as well as the daily logistics of moving the teams.

### Baseline census

The full baseline census was conducted in the entire surveillance area after the initial pilot. The census was conducted between September and December 2010. The full baseline population of the surveillance area was 125,149 from 19,193 households. The methodology, results, and findings from the Nahuche HDSS full baseline have been reported elsewhere (10).

### Surveillance rounds

After the baseline census, six-monthly cycles of data collection beginning in January 2011 were conducted in all households identified during the baseline census and new households were also registered. Trained interviewers visited the communities under surveillance, record events (pregnancies, births, deaths, migration, marriages, and vaccination coverage) in registers, and report data to the Nahuche HDSS Computer Centre for processing. As of December 2012, the population under surveillance was 144,496 residing in 20,371 households (average of seven individuals per household).

For each round, additional elements of the surveillance system have been put into place. At Round 1, we began the listing of movements in and out of the households, so that migrations could be tracked into and out of the surveillance community. With Round 2, we designated in-migrants and out-migrants depending on their change of status from Round 1 to Round 2. We also added a module on the maternal and child health, including more details about immunisations. At Round 4 and not Round 3, we added verbal autopsy methods, joining the other INDEPTH sites which are trained to inquire about the circumstances just before and at the time of death for any deaths occurring between the rounds. These verbal autopsies are submitted to a panel of three trained physicians who code the presumed cause of death according to the signs and symptoms noted.

### Sustainability and the 5-year strategic plan

Stakeholders developed a strategic planning framework for Nahuche HDSS, which includes the mission, vision, and objectives for the 5-year period beginning from 2013. The context within which the strategies were selected for each priority area was based on the SWOT analysis. The priority areas of the framework are: strengthening institutional management; human resource development; sustainable funding; infrastructure development; data quality and INDEPTH Network Membership registration (obtained in November 2012); research agenda; community engagement, support, and benefit; dissemination and communication of findings; and partnership and networking.

The strategic plan has since been implemented and institutional management arrangements are being put in place for the centre. The budget for the 5-year plan defines the expected sources of funding of the various components of the plan. Collaborators from UDUS, our international advisors, and the state government are leading this process.

## Discussion

### How we met the challenges encountered in establishing an INDEPTH HDSS site

#### Stakeholders’ consultation and sustainability

Stakeholders’ consultation and participation is key to successful implementation of longitudinal population surveillance. At the inception, major stakeholders in the Ministries of Health, Budget and Economic Planning, traditional leaders, and communities were involved during the setting up of the centre, and many details needed to be resolved in a timely fashion. Therefore, stakeholders’ meetings were held monthly in order to ensure a rapid response to the emerging and multiple operational issues concerning setting up the HDSS. The meetings became less frequent as significant progress was made.

At the recommendation of the stakeholders for a strategy to ensure the Nahuche HDSS sustainability beyond the lifetime of the PRRINN-MNCH Programme, two MoUs were signed. The first MoU was signed between the PRRINN-MNCH Programme and the Zamfara State government to address the human resource needs as well as the financial needs of the centre beyond the programme's life span. Another MoU was subsequently signed between the Zamfara State government and UDUS for the latter to lead all the technical and scientific processes at the centre. Part of the technical leadership involves resource mobilisation through application for grants through proposal development from international agencies and overall technical management of the centre. UDUS has also incorporated part of the staff of the Nahuche HDSS into the UDUS human resources structures.

HDSS stakeholder consultations also benefitted from the influence of the strong traditional leadership system that facilitated obtaining the maximum required cooperation from the people. This has been complemented from time to time by holding mobilisation meetings with the traditional and community leaders.

#### Recruitment of staff

Human resource constraints are part of the serious challenges in northern Nigeria. Nahuche is an impoverished, rural community, which makes it ideal for examining the impact of interventions, but poses challenges for recruiting women interviewers, needed due to cultural sensitivity but in limited supply due to low literacy levels, especially among women. We successfully recruited women through a variety of innovations to the usual recruitment process. We sought recommendations from LGA and community leaders of organisations or even individuals who would make good interviewers even if they did not meet the minimum literacy standards. We also recruited through the National Youth Service Corps Programme of the Federal Government of Nigeria. We identified female corps members fluent in Hausa, and they were trained according to the operational guidelines for HDSS fieldwork, as well as in the basic cultural norms and values of the communities. This approach had several advantages. The advantages for the HDSS include more complete coverage through lower refusal or non-completion rates by female respondents and higher quality data, due to their level of education and training, which enabled them to be effective interviewers and to complete the forms properly. Further, the HDSS had the benefit of the corps members at lower cost, since the corps members were paid only monthly allowances, as their program covered their stipends. For the corps members, working at the HDSS afforded them an opportunity to add more value to their academic status, along with additional income. Because they like the work, some corps members were retained after their mandatory 1 year service.

#### Retention of staff

A perennial problem for the Nahuche HDSS is the retention of staff. We have had difficulties retaining data entry clerks, who work in a confined space entering data, day in and day out. The conditions of work are difficult, and staff have asked twice for higher pay. Others have simply failed to meet their quota of interviews per day. The Nahuche HDSS has responded with slight increases in pay, but more importantly has put into place a performance incentive that rewards the interviewers upon meeting standards slightly above the expected target. Supervisors have also been trained in supportive supervision, so that they are less judgemental and more oriented to helping the interviewers solve problems. Retention of staff has been further addressed by making all HDSS staff affiliates of the UDUS.

Retention of female staff remains a problem, however, as the demands of the fieldwork are rigorous, spending many hours in the community with limited access to water or sanitation facilities. In addition, we have lost female interviewers due to resignations when they marry or move with their husbands.

#### Community mobilisation

Longitudinal population surveillance is new in Nigeria. Community acceptance of this innovation requires constant social mobilisation and community engagement. Such community engagement is vital to the continued acceptability of our team which includes some male interviewers who must speak with women about births and other maternal and child health details. There is religious hostility towards some of the PRRINN interventions, such as immunisation and family planning, which are seen as foreign to their culture. Even though the interviewers are not linked directly to these programmes, they are seen as associated with it. The challenge has been to continue to elicit participation when not everyone is experiencing benefits from the programme. Some of the programme's interventions operate through the PHC system, and if the householder has not visited the system, they may be unaware of the programme. Similarly, not all of the community interventions are equally present in these communities, and women asked about community health workers may have had no contact with them. Poverty remains pervasive, and the interviewers offer no incentives for the time that families take for the interviews every 6 months.

In order to retain high interest and support for the semi-annual interview process, HDSS implemented a continuous advocacy campaign to build a strong community–HDSS relationship. The Nahuche HDSS team assembled a 30-member community mobilisation committee, five in each district. The responsibilities of the committee include advocacy and sensitisation campaigns and mediating roles during any form of misunderstanding between the community and the HDSS. Most times, the committee advises the HDSS management on how best to avoid confrontation. Whenever a community rejected the interviewers, interviewing was immediately stopped, and the district mobilisation committee launched an intense sensitisation and advocacy campaign, including community dialogue, use of town criers, and meeting with the village heads and household heads.

Longitudinal surveillance is often characterised by respondent fatigue, and to counter this threat, the HDSS created an incentive for the community participation, namely the opportunity to participate in implementation research through which they would receive specific health services not widely available. Rather than describing it as a passive study that was being done with no benefit to them, these studies offer concrete assistance to families in the intervention zones of the HDSS. The first collaboration has been with a nutritional surveillance and intervention project. All women in the selected intervention districts within the HDSS are invited to participate in community dialogues to learn more about how to offer their children a balanced diet, particularly using local foods. During their routine visits, the HDSS fieldworkers identify children with acute malnutrition by measuring the middle upper arm circumference of all children between 6 and 59 months. Based on the criteria for acute malnutrition from this measurement, they refer those with acute malnutrition to the sister project for urgent medical and nutritional intervention. A second collaboration is to pilot the use of misoprostol to stop post-delivery bleeding and chlorhexidine to reduce infections to the umbilical cord. These are given to women at 8 months of pregnancy and at the onset of labour by trained community-based service delivery Community Health Extension Workers (CBSD CHEWs) since most deliveries are conducted at home within surveillance area, and also available to women coming to the Nahuche PHC for their deliveries. Thus, the implementation research studies, the raison d'etre for the HDSS, themselves become part of the positive community mobilisation for participation in the HDSS.

#### Security

There have been a number of worrisome security-related developments in Nigeria's recent history. From the 2011 post-election violence in some northern parts of the country to the ongoing insurgency of the Islamic extremist group known as *Boko Haram*, the recurrent violence and insecurity poses a great challenge to maintaining the high standards of data collection required for the Nahuche HDSS. For example, international partners, including members of the INDEPTH Network, are not allowed to travel to Zamfara and other parts of northern Nigeria. Thus, planned activities are cancelled most of the time or the venue for such activities is shifted to either outside the country or the Federal Capital Territory, Abuja. The implications of either the total cancellation of scheduled activities or change of activities’ venue ranges from disrupted work plans to high operational costs.

The approach to resolving this challenge requires changing the venue of meetings to Abuja or Lagos to allow international partners to participate. Instead of INDEPTH trainers and support coming to the Nahuche HDSS, HDSS staff have gone to the trainers for training. For example, training on verbal autopsy data collection and management was held at the Kintampo HDSS in Ghana, while the database development training for the data manager was held at the Navrongo HDSS in Ghana.

## Conclusion

Even though the INDEPTH Network has clear guidelines and a programme of technical support to help researchers develop health and demographic surveillance sites, our experience in developing the Nahuche HDSS shows that it was very important to be able to adapt the guidelines to the particular needs and situation of the selected site. In the case of the Nahuche HDSS, our greatest resource and the reason Nahuche was selected was that the community provided an opportunity to conduct implementation studies in a setting with a very marginalised and vulnerable population, and one with very strong Muslim and local cultural traditions. Thus, what we learn in Nahuche is valuable for much of northern Nigeria and neighbouring Sahelian countries with similar populations and traditions. But these very qualities which make Nahuche valuable as a surveillance site also have posed the greatest challenges to the establishment of the HDSS site. The low literacy levels, particularly among women, made it difficult to recruit female interviewers, required to be consistent with local customs of gender separation and isolation. The solutions involved changes to recruitment and retention practices that bent the rules and brought in national service corps volunteers. Not only did this solve the gender balance problem for the team, their participation in the HDSS fieldwork team helped these women with their own personal advancement goals. The antipathy to the health care system, a common barrier that health system reform seeks to overcome, was countered by strong community mobilisation actions. Instead of passively seeking the approval of community residents for participation studies ‘conducted’ on the HDSS community, their participation was solicited and introduced as evidence of the programme's gratitude to their willingness to participate in the HDSS routine interviews. Problems with recruiting senior staff have been resolved through developing a strong partnership with a nearby university. Sustainability of the HDSS has been addressed through Memoranda of Understanding with Zamfara State and the UDUS, which share responsibility for the governance and future of the HDSS as an implementation research centre.

Not that all is smooth sailing. Much remains to be done to ensure sustainability. There are still gaps in the scientific leadership of the HDSS, which need to be filled by UDUS. Zamfara State has not actually released the funds for supporting the HDSS, and advocacy visits need to be organised for this purpose. Various infrastructure improvements have yet to be accomplished.

While the experiences of setting up a new HDSS site may vary across settings, the experiences in northern Nigeria offer some strategies that may be replicated in other settings with similar challenges. While issues related to lack of community support and ownership can be resolved with continuous community mobilisation, ensuring that stakeholders and funding partners such as governments commit to their tasks is one of the greatest challenges we have encountered. Nevertheless, continuous engagement and sensitisation of all stakeholders is a critical step in ensuring sustainability. We hope that the experiences documented here will provide opportunities to refine strategies for setting up an HDSS in similar settings.
